# Shifts in Soil Microbial Community Composition, Function, and Co-occurrence Network of *Phragmites australis* in the Yellow River Delta

**DOI:** 10.3389/fmicb.2022.858125

**Published:** 2022-07-19

**Authors:** Pengcheng Zhu, Shuren Yang, Yuxin Wu, Yuning Ru, Xiaona Yu, Lushan Wang, Weihua Guo

**Affiliations:** ^1^Key Laboratory of Ecological Prewarning, Protection and Restoration of Bohai Sea, School of Life Sciences, Ministry of Natural Resources, Shandong University, Qingdao, China; ^2^State Key Laboratory of Microbial Technology, Shandong University, Qingdao, China

**Keywords:** Yellow River Delta, soil, bacteria, fungi, *Phragmites australis*, inter-kingdom microbial co-occurrence network

## Abstract

Soil microorganisms play vital roles in regulating biogeochemical processes. The composition and function of soil microbial community have been well studied, but little is known about the responses of bacterial and fungal communities to different habitats of the same plant, especially the inter-kingdom co-occurrence pattern including bacteria and fungi. Herein, we used high-throughput sequencing to investigate the bacterial and fungal communities of five *Phragmites australis* habitats in the Yellow River Delta and constructed their inter-kingdom interaction network by network analysis. The results showed that richness did not differ significantly among habitats for either the bacterial or fungal communities. The distribution of soil bacterial community was significantly affected by soil physicochemical properties, whereas that of the fungal community was not. The main functions of the bacterial and fungal communities were to participate in the degradation of organic matter and element cycling, both of which were significantly affected by soil physicochemical properties. Network analysis revealed that bacteria and fungi participated in the formation of networks through positive interactions; the role of intra-kingdom interactions were more important than inter-kingdom interactions. In addition, rare species acted as keystones played a critical role in maintaining the network structure, while ${\mathrm{NO}}_{3}^{-}-N$ likely played an important role in maintaining the network topological properties. Our findings provided insights into the inter-kingdom microbial co-occurrence network and response of the soil microbial community composition and function to different *P. australis* habitats in coastal wetlands, which will deepen our insights into microbial community assembly in coastal wetlands.

## Introduction

Soil microorganisms, the main decomposers of plant and animal residues, are important components of the ecosystem and play key roles in regulating biogeochemical processes, including carbon, nitrogen, and sulfur cycling (Madsen, 2011). Soil microbial community diversity and composition are susceptible to variations in biotic and abiotic factors, including plant litter, soil physicochemical properties, and climatic factors (Cavicchioli et al., 2019; Gołębiewski et al., 2019; Duan et al., 2021). In particular, soil is a direct medium for the life of soil microorganisms, and slight changes in its physicochemical properties will significantly affect the diversity and composition of soil microorganisms. For instance, soil pH is the best predictor of the global distribution of bacteria and archaea (Fierer, 2017), and soil salinity drives the distribution pattern of fungal communities in coastal wetlands (Yang and Sun, 2020). Furthermore, in terms of soil microbial biodiversity and biomass, both bacteria and fungi dominate the soil habitat, and their community compositions in response to soil physicochemical properties affect the overall ecosystem functions (Liu et al., 2020; Chen et al., 2020a). Therefore, a comprehensive and in-depth study of the diversity, composition, and function of soil bacterial and fungal communities, rather than the response of a single bacterial or fungal community, to habitats with heterogeneous soil physicochemical properties, is helpful for assessing changes in ecosystem functions.

In the natural environment, microorganisms associate with each other through complex interactions (Zhang et al., 2018; Zhao et al., 2020; Qian et al., 2021). Network analysis, as an effective tool for evaluating microbial interactions, has been widely used to construct microbial co-occurrence networks in different habitats, such as soil and marine habitats (Lima-Mendez et al., 2015; Zhang et al., 2018). Although two significantly associated nodes of the microbial network may not always account for the correct biological interactions, network analysis can help understand the complexity of microbial communities and how microbial community complexity responds to environmental variation (Qiu et al., 2021). The complexity of the microbial network is embodied in parameters reflecting connectivity among microorganisms, such as degree, modularity, and clustering coefficient, which are significantly influenced by environmental factors (Ma et al., 2016; Zhang et al., 2018). The connectivity of microbial networks is considered to be a key factor in the stability of the microbial community and ecosystem multifunctionality, and nodes with multiple connections are considered as keystone taxa maintaining the microbial network structure (Shi et al., 2016; Qian et al., 2021). Evaluating the key factors affecting the connectivity indices and identifying the keystone taxa of the microbial network can deepen our understanding of microbial community construction (Wagg et al., 2019). Additionally, most recent studies have focused on microbial intra-kingdom interactions while ignoring the importance of microbial inter-kingdom interactions. However, previous studies have proven the ubiquity of microbial inter-kingdom interactions, especially between bacteria and fungi; for example, fungi secrete antimicrobial compounds to outcompete bacteria, and lignocellulase, which cooperates with bacteria to degrade recalcitrant carbohydrates (Zhang et al., 2015; Bahram et al., 2018). Thus, it is essential to evaluate inter-kingdom interactions between microorganisms (bacteria, fungi, archaea, etc.; Cheung et al., 2018; Zhu et al., 2021b).

Coastal wetlands, as interactive zones between freshwater and seawater, are strongly influenced by both ocean and land, and they have complex and changeable environment. The high biodiversity of coastal wetlands play a key role in maintaining ecological functions (Osland et al., 2018). The Yellow River Delta, a young, expanding, and strongly distributed coastal wetland, contains a variety of habitats, provides multiple ecological services, and has attracted increasing attention for its irreplaceable and pivotal ecological values. Recent investigations of the microbial community in the Yellow River Delta have primarily focused on microbial diversity, composition, and their controlling factors under different plant communities, salinity, and human disturbance, while ignoring the effects of different habitats on the same plant (Gao et al., 2021; Wu et al., 2021). *P. australis* is a common species in coastal wetlands because of its unique and high reproductive capacity and environmental adaptability; it is widely distributed in various habitats in the Yellow River Delta (Liu et al., 2021). Chi et al. (2021) evaluated the soil bacterial community composition and function of *P. australis* in different habitats and highlighted the key role of soil organic matter and salinity in affecting bacterial composition and function, but neglected the response of fungal communities to different habitats, resulting in a lack of effective information to evaluate the inter-kingdom interactions of soil microorganisms in *P. australis*.

In this study, we measured the soil bacterial and fungal community composition in five different *P. australis* habitats, constructed an inter-kingdom microbial co-occurrence network, and investigated their controlling factors. The specific goals of this study were to: (1) investigate the diversity, composition, and potential function of soil bacterial and fungal communities in different *P. australis* habitats; (2) reveal the potential interactions of soil microorganisms containing bacteria and fungi based on network analysis and evaluate the keystone taxa of the microbial networks; and (3) identify the controlling factors of microbial community composition, function, and microbial interactions.

## Materials and Methods

### Study Area Description and Soil Sampling

**Figure 1:**
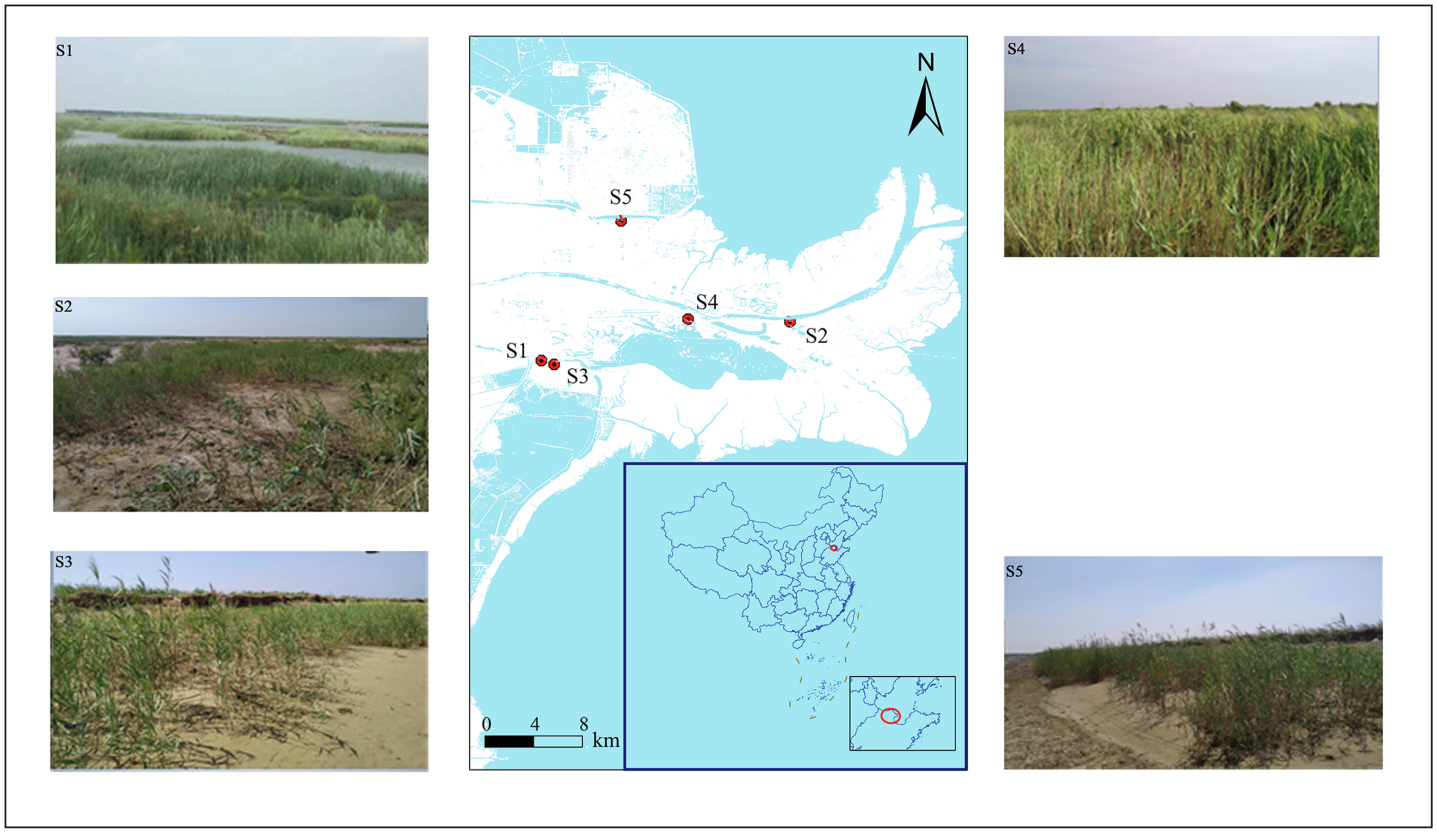
Sampling site locations in the Yellow River Delta.

This study was conducted in the Yellow River Delta, Shandong Province, China (118°33′E–119°20′E, 37°35′N–38°12′N) in 2019. This region has a temperate monsoon climate with an annual average temperature of 12.1°C and 196 frost-free days. The average annual precipitation is 530–630 mm, of which 70% is concentrated from June to August. The soil types are latent regional fluvial and saline. Five typical *P. australis* habitats were selected for soil sampling. Detailed information about habitat location is shown in Figure 1 and Supplementary Table S1. Surface soil sampling and plant community surveys were conducted in August, when *P. australis* was the most vigorous. At each of the three repeat sampling locations, five subsamples of topsoil (0–15 cm) were collected after removing the topsoil plant litter and thoroughly mixed to obtain a representative topsoil sample. We collected 15 samples, which were immediately stored in an ice box and transported to the laboratory. Each homogenised sample was divided into two parts: one was stored at −20°C for further microbial DNA extraction, and the other was air-dried to analyse its physicochemical properties.

### Soil Physicochemical Property Analysis

The air-dried soil samples were filtered through a 2 mm mesh screen to remove visible plant roots and pebbles. The pH value of the water extract of each sample was determined using a distilled water ratio of 1:5 (w/v) and a pH metre (FiveEasy Plus^™^, Mettler Toledo, Switzerland). The same water extract was used to determine the electrical conductivity (EC; FiveEasy Plus^™^, Mettler Toledo, Switzerland). Soil organic matter (OM) content was determined using the potassium dichromate volumetric method. Based on the method provided by Zhu et al. (2021a), the total nitrogen (TN) and total phosphorus (TP) contents of the soil samples were determined using the Kjeldahl method and molybdenum antimony colorimetry, respectively. The soil extract extracted with a 2 M KCl solution was used to determine nitrate nitrogen and ammonium nitrogen using a continuous flow analyser (San^++^ SYSTEM, Skalar, Netherlands). Available potassium (AK) was extracted by 1 M ammonium acetate solution and subsequently measured based on the atomic absorption spectrum.

### DNA Extraction and High-Throughput Sequencing

The total DNA of the soil sample was extracted from a 0.500 g soil sample using the DNA Soil Kit (TIANGEN BIOTECH, Beijing, China) according to the manufacturer’s instructions. The quality and concentration of DNA were determined using a NanoDrop^®^ ND-1000 Spectrophotometre (Thermo Fisher Scientific Inc., Waltham, MA, United States) and 1.0% agarose gel electrophoresis. The DNA was stored at −20°C until further processing. Using the Illumina MiSeq platform by Majorbio Biotech. Co., Ltd., bacterial and fungal communities were assessed using 16S rRNA gene and internal transcribed spacer (ITS) high-throughput sequencing, respectively. Non-conserved 16S V3V4 regions were amplified using primers 341F (5-ACTCCTACGGGAGGCAGCAG-3) and 806R (5-GGACTACHVGGGTWTCTAAT-3), whereas ITS1F (5-CTTGGTCATTTAGAGGAAGTAA-3) and (5-GCTGCGTTCTTCATCGATGC-3) were used to amplify the ITS regions. To improve the quality of downstream analysis, the QIIME pipeline (version 1.7.0) was used to perform sequence stitching and quality trimming of raw data from Illumina MiSeq sequencing (Caporaso et al., 2010). After quality filtration, clean reads were clustered to generate operational taxonomic units (OTUs) with a 97% similarity identity cut-off using Uparse (version 7.0.1090). The most abundant sequence in each OTU, taken as a representative sequence, was selected to annotate taxonomic level using RDP classifier (version 2.11) based on the Silva database (Version 138). Unidentified and non-microbial sequences were defined as ‘unclassified’ for subsequent analysis.

### Definition of Rare and Abundant Taxa

According to Xu et al. (2021), based on the relative abundance and occurrence frequency of OTUs in samples, they were divided into six classifications: always abundant taxa (AAT) with relative abundance >1% in all samples, conditionally abundant taxa (CAT) with relative abundance >1% in some samples and never <0.01%, always rare taxa (ART) with relative abundance <0.01% in all samples, conditionally rare taxa (CRT) with relatice abundance <1% in all samples and <0.01% in some samples, moderate taxa (MT) with relative abundance between 0.01 and 1% in all samples, and conditionally rare and abundant taxa (CRAT) with relative abundance ranging from rare (< 0.01%) to abundant (>1%). Among these classifications, AAT, CAT, and CRAT were considered abundant taxa, whereas ART and CAT were regarded as rare abundant taxa.

### Network Analysis and Visualisation

Based on the R (Version 4.0.2) software package ‘Hmis’, the correlation matrix of microbial community was obtained by calculating the Spearman correlation coefficient between bacterial and fungal OTUs detected in more than eight samples. The false discovery rate (FDR) method was used for multiple testing corrections of the generated *p*-values (Yoav and Yosef, 1995). Significantly related OTUs (*p* < 0.05, *r* > 0.8) were used to construct a co-occurrence network graph. Co-occurrence of the microbial community was visualised using Gephi (version 0.9.2). Furthermore, the network characteristics were calculated using Gephi. The topological role of each node was assessed using the threshold values of Within-module connectivity (*Zi*) and Among-module connectivity (*Pi*), as proposed by Zhu et al. (2021a). Moreover, based on topological properties of sub-networks calculated by the ‘subgraph’ function, we evaluated the correlations between soil physicochemical properties and topological properties of the network.

### Statistical Analysis

Statistical analysis and figures were carried out in R (Version 4.0.2). One-way analysis of variance (ANOVA) was conducted using the ‘agricolae’ package to assess the discrepancy of microbial alpha diversity and soil physicochemical properties. Based on the ‘vegan’ package, principal coordinate analysis (PCoA) and permutational multivariate analysis of variance (PERMANOVA) were conducted to visualise and digitise the dissimilarity in microbial community composition and function, respectively. Subsequently, the histograms and heatmaps were performed using the ‘ggplot2’ and ‘pheatmap’ packages, respectively. Procrustes analysis was applied to explore the correlations between the microbial community and soil physicochemical properties (TN, TP, OM, ${\mathrm{NH}}_{4}^{+}-N$ , ${\mathrm{NO}}_{3}^{-}-N$ , AK, pH, and EC) in the ‘vegan’ package. A chord diagram showing the distribution of links among the bacterial and fungal phyla in co-occurrence network was constructed using the ‘circlize’ package. Canonical correspondence analysis (CCA) was performed using the ‘vegan’ package. In addition, based on the Majorbio I-Sanger Cloud Platform^1^ designed for bioinformation analysis, we predicted bacterial and fungal community functions using PICRUST2 and FUNGuild tools. PICRUST2 classifies bacterial community functions according to the metabolic pathways of bacterial genes in KEGG (Douglas et al., 2020). FUNGuild is an open annotation tool for parsing fungal community datasets by ecological guild (Nguyen et al., 2015). Similarly, linear discriminant analysis (LDA) combined with linear discriminant analysis effect size (LEfSe) was conducted to identify the biomarker microorganisms based on the cloud platform.

## Results

### Physicochemical Properties of Soil Samples

*Phragmites australis* is distributed in different habitats with different physicochemical properties in the Yellow River Delta. As shown in Table 1, most soil physicochemical indices exhibited significant differences among the five soil samples, except ${\mathrm{NH}}_{4}^{+}-N$ (*p* > 0.05). As the dominant stress factors affecting plant community in saline-alkali land, the values of pH and EC of soil in different *P. australis* ranged from 7.65 to 8.07 and 1.27 to 5.36 mS/cm, respectively. Among the soil nutrient indices, OM, TN, ${\mathrm{NH}}_{4}^{+}-\mathrm{N,}$ ${\mathrm{NO}}_{3}^{-}-\mathrm{N,}$ and TP ranged from 17.24 to 58.99 mg/g, 70.00 to 169.79 mg/kg, 3.08 to 3.81 mg/kg, 1.14 to 5.41 mg/kg and 1.01 to 1.36 mg/g, respectively.

**Table 1:** Physicochemical properties of soil samples.

Sample	pH	EC (mS/cm)	OM (mg/g)	TN (mg/kg)	$NH_{4}^{+}-N$ (mg/kg)	$NO_{3}^{-}-N$ (mg/kg)	TP (mg/g)
S1	7.88 ± 0.11a	3.09 ± 1.44b	42.14 ± 4.35b	70.00 ± 18.12c	3.11 ± 0.10a	5.41 ± 0.82a	1.03 ± 0.05c
S2	7.99 ± 0.15a	1.27 ± 0.82b	58.99 ± 5.43a	104.67 ± 21.07bc	3.08 ± 0.61a	1.30 ± 0.50b	1.01 ± 0.05c
S3	7.65 ± 0.19b	5.36 ± 1.89a	27.58 ± 1.99c	127.67 ± 6.17ab	3.81 ± 0.60a	1.18 ± 0.20b	1.36 ± 0.01a
S4	8.04 ± 0.02a	1.53 ± 0.90b	22.41 ± 4.70 cd	160.22 ± 41.94a	3.31 ± 0.27a	1.75 ± 0.67b	1.09 ± 0.06bc
S5	8.07 ± 0.08a	1.45 ± 0.27b	17.24 ± 3.04 cd	169.79 ± 36.99a	3.25 ± 0.56a	1.14 ± 0.40b	1.20 ± 0.10b

Data represent the average and standard deviation of three values. Data followed by the same letter are not significantly different ANOVA, *p* < 0.05.

### Characteristic of Soil Microbial Community

**Figure 2:**
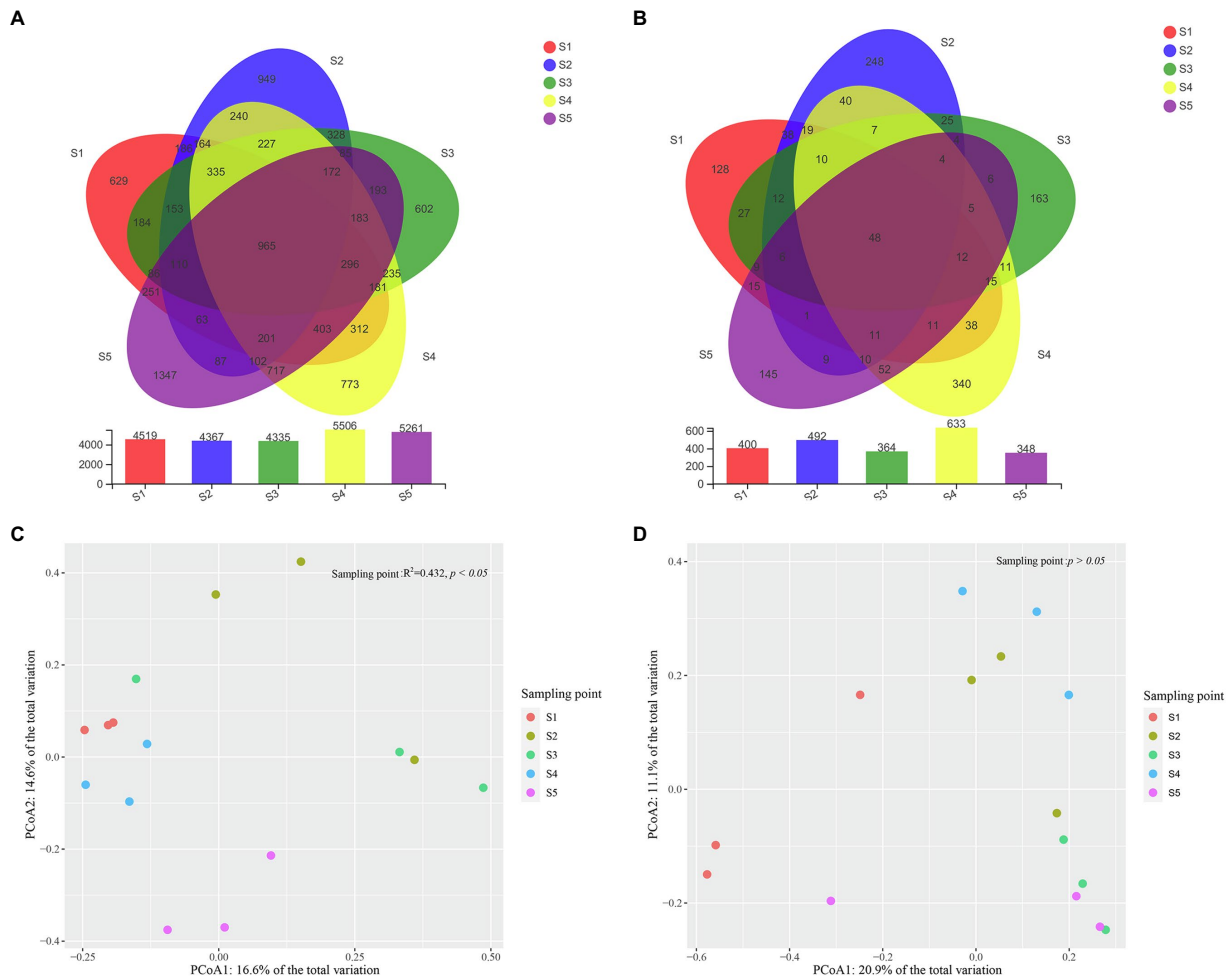
Characteristics of microbial community compositions. Shared bacterial **(A)** and fungal **(B)** operational taxonomic units (OTUs) across different samples. Principal components analysis (PCoA) of bacterial **(C)** and fungal **(D)** community composition calculated with Bray–Curtis distances. The results of Permutational multivariate analysis of variance analysis (PERMANOVA) show a significant association of bacterial community composition with different *P. australis* habitats (^*^p < 0.05).

In total, 512,759 high-quality bacterial reads and 615,503 high-quality fungal reads were obtained, after removing potential chimeras. Based on 97% sequence similarity, the bacterial and fungal sequences were clustered into 11,299 and 1,635 OTUs, respectively. The rarefaction curve of bacterial and fungal OTUs reached the saturation platform, which suggested that most of the bacterial and fungal taxa in the soil samples were detected (Supplementary Figure S1). The number of bacterial and fungal OTUs detected in each *P. australis* habitat ranged from 4,335 to 5,506 and 348 to 633, respectively, and the number of shared bacterial and fungal OTUs among *P. australis* habitats was 965 and 48, respectively (Figures 2A,B).

Alpha diversity indices (Shannon index and Chao1) are important indicators reflecting the distribution patterns of species. The observed species, Shannon and Chao1 indices of bacterial community among five habitats ranged from 845.33 to 1.302, 4.72 to 5.68, and 988.70 to 1532.15, respectively. Regarding the fungal community observed species, Shannon and Chao1 indices ranged from 150.33 to 280.67, 2.26 to 3.57 and 165.72 to 301.54, respectively (Table 2). There were no significant differences in the observed species numbers, Shannon index, or Chao1 index of both the bacterial and fungal communities among the five *P. australis* habitats (ANOVA, *p* > 0.05).

**Table 2:** α-diversity indices of the microbial communities.

Bacterial index			Fungal index		
	Observe species	Shannon	Chao1	Observe species	Shannon	Chao1
S1	1182.3 ± 56.89a	5.57 ± 0.06a	1385.58 ± 35.34a	201.00 ± 53.83a	2.26 ± 0.94a	241.70 ± 35.07a
S2	981.33 ± 278.68a	5.14 ± 0.60a	1109.25 ± 227.58a	207.00 ± 37.99a	2.98 ± 0.92a	230.58 ± 50.23a
S3	845.33 ± 328.89a	4.72 ± 1.12a	988.70 ± 339.35a	162.00 ± 58.64a	3.13 ± 0.92a	175.49 ± 62.60a
S4	1302.33 ± 134.92a	5.68 ± 0.18a	1532.15 ± 113.17a	280.67 ± 99.01a	3.57 ± 0.62a	301.54 ± 104.53a
S5	1,085 ± 154.24a	5.60 ± 0.17a	1259.14 ± 190.13a	150.33 ± 19.14a	3.38 ± 0.37a	165.72 ± 11.23a

Data represent the average and standard deviation of three values. Data followed by the same letter are not significantly different ANOVA, *p* < 0.05.

A PCoA based on Bray–Curtis distance metrics was constructed to depict the differences in microbial communities among the *P. australis* habitats (Figures 2C,D). Coordinate axes 1 and 2 explained 16.9 and 14.5% of the variation in the bacterial community and 20.9 and 11.1% of the variation in the fungal community, respectively. This showed that there were clear bacterial and fungal distribution patterns in different *P. australis* habitats. PERMANOVA indicated that the bacterial communities of the five *P. australis* habitats were significantly different from each other (*R^2^* = 0.429, *p* < 0.05), while the whole fungal community showed no significant difference (*R^2^* = 0.314, *p* > 0.05), indicating that different habitats of *P. australis* have a significant influence on the bacterial community but not on the fungal community.

### Microbial Community Composition

**Figure 3:**
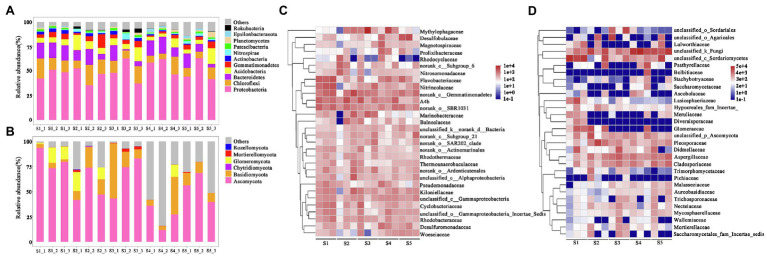
Relative abundances of microbial community compositions in each sample. **(A)** and **(B)** represent the relative abundances of bacterial and fungal phylum, respectively. **(C)** and **(D)** represent the relative abundances of the top 30 bacterial and fungal genera, respectively.

After classification analysis of 16S rRNA and ITS genes sequences, a total of 57 bacterial phyla and 9 fungal phyla were identified. The five dominant bacterial phyla (Proteobacteria, Chloroflexi, Bacteroidetes, Acidobacteria, and Gemmatimonadetes) accounted for 76.8–90.3% of the identified bacterial sequences (Figure 3A). Among them, Proteobacteria had the highest relative abundance in the *P. australis* soil, followed by Chloroflexi or Bacteroidetes. Bacteroidetes with the second highest relative abundance in S1 and S4, while Chloroflexi had the second highest relative abundance in S2, S3, and S5. For fungal community, dominant Ascomycota and Basidiomycota, representing 16.5–98.7% of identified fungal sequences within 15 soil samples (Figure 3A). Among them, Ascomycota was found to have the highest relative abundance in all the soil samples, with relative abundances ranging from 11.8 to 96.3% (Figure 3B).

At the family level, we focused on the responses of bacterial and fungal families with a top 30 relative abundance in *P. australis* habitats (Figures 3C,D). The top 30 bacterial families accounted for 30.0–65.4% of the total sequences, in which 15 families belong to Proteobacteria, 5 families belong to Bacteroidetes, 4 families belong to Acidobacteria, and 4 families belong to Chloroflexi. In terms of average relative abundance, the most predominant bacterial family was Methylophagaceae, with an average value of 4.7%, followed by norank_class__Gemmatimonadetes (4.0%), A4b (3.8%), norank_order__SBR1031 (3.2%), Flavobacteriaceae (3.0%), Nitrincolaceae (2.5%), Desulfobulbaceae (2.4%), and Kiloniellaceae (2.0%). Similarly, the top 30 fungal families accounted for 78.1–99.7% of the total sequences, in which 18 families belong to Ascomycota and 8 families belong to Basidiomycota. The most predominant family was unclassified_kingdom_fungi (23.3%), followed by unclassified_class__Sordariomycetes (17.9%), Pleosporaceae (5.9%), Aspergillus (5.6%), unclassified_order_Agaricales (5.3%), Glomeraceae (4.4%), and Cladosporiaceae (4.0%).

LEfSe tools were used to investigate biomarkers that caused differences among different *P. australis* habitats (Supplementary Figure S2). The results showed that 21 bacterial clades exhibited significant differences in different *P. australis* habitats with an LDA threshold of 3.5, whereas the number of fungal clades was 20 (Supplementary Figures S2C,D). In each *P. australis* habitat, the significantly enriched bacterial and fungal taxa varied. Among them, the number of bacteria and fungi enriched in the S5 plot was the largest, at 9 and 12, respectively. Moreover, no enriched fungal taxa were detected in S4.

### Predicted Functions of Microbial Communities

**Figure 4:**
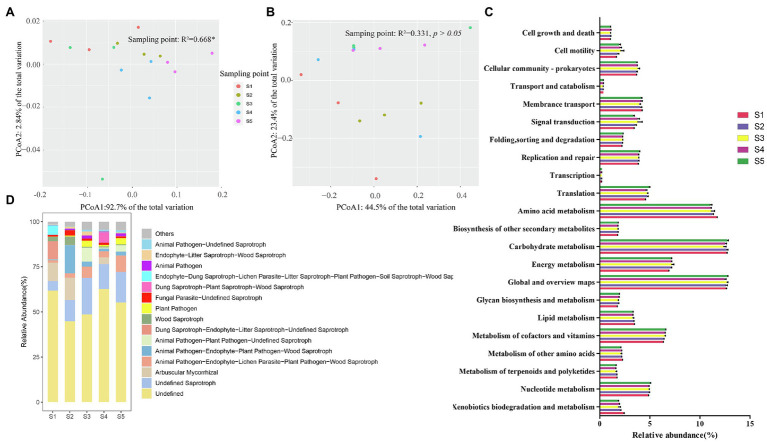
Predicted function of microbial community. Discrepancy **(A)** and composition (**C**) of bacterial community function predicted based on PICRUSt2. Discrepancy **(B)** and composition **(D)** of fungal community function predicted based on FUNGuild.

The functional characteristics of the soil bacterial and fungal communities in different *P. australis* habitats were predicted using PICRUSt2 and FUNGuild, respectively (Figure 4). In bacterial community function, six categories at level 1 containing metabolism (65.7–68.7%), genetic information processing (10.6–11.6%), environmental information process (7.1–8.5%), cellular processes (6.5–8.2%), human diseases (4.0–4.8%), and organismal systems (2.0–2.2%) were identified (Figure 4C). At level 2, 45 pathways were identified, of which most abundant functional categories were global and overview maps (12.2–12.9%), carbohydrate metabolism (11.9–12.9%), amino acid metabolism (10.4–11.7%), energy metabolism (6.8–7.6%), and metabolism of cofactors and vitamins (6.3–6.6%). PCoA based on the Bray–Curtis distance revealed that different *P. australis* habitats significantly affect soil bacterial community function (PERMANOVA, *p* < 0.05; Figure 4A).

The FUNGuild results showed a total of 68 guilds belonging to 8 trophic modes: saprotrophs, saprotroph-symbiotrophs, pathotroph-saprotrophs, pathotrophs, symbiotrophs, pathotroph-saprotroph-symbiotroph, pathotroph-symbiotroph, pathogen-saprotroph-symbiotroph, and pathogen-saprotroph-symbiotroph (Figure 4D). Generally, the information on fungal species data is far less than that of bacterial data, resulting in a large amount of fungal OTU classification information that has not been identified. Among the identified fungal guilds, undefined saprotrophs were the most predominant guild (mean relative abundance accounting for 13.5% of the total relative abundance), followed by animal pathogen-endophyte-plant pathogen-wood saprotrophs (5.6%), arbuscular mycorrhizal (4.8%), animal pathogen-endophyte-lichen parasite-plant pathogen-wood saprotrophs (4.3%), and animal-pathogen-plant pathogen-undefined saprotrophs (2.2%), among others. Similar to the discrepancy in microbial community composition, the function of the soil fungal community was not sensitive to *P. australis* habitats (PERMANOVA, *p* > 0.05; Figure 4B).

### Topological Properties of the Co-occurrence Network

**Figure 5:**
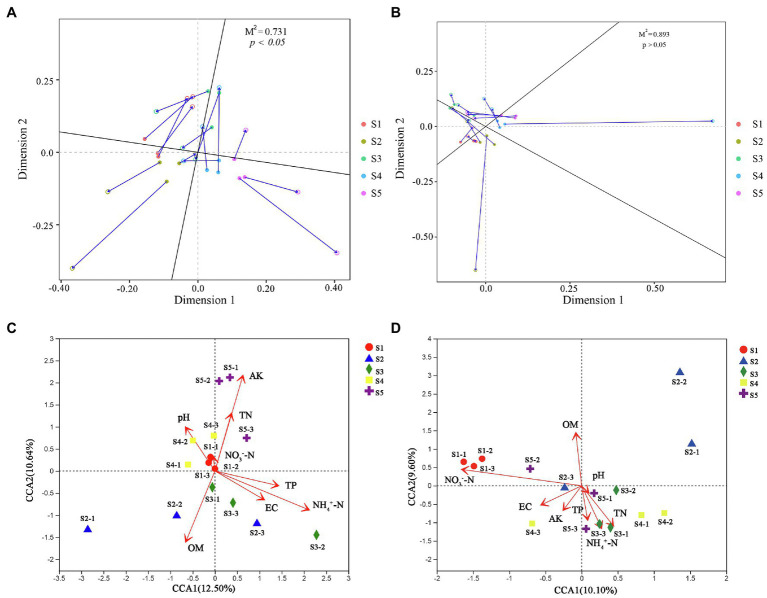
Soil microbial community affected by soil physicochemical properties. Procrustes analysis evaluating the relationships between soil physicochemical properties and bacterial **(A)** and fungal **(B)** communities. Redundancy analysis (RDA) of the bacterial **(C)** and fungal **(D)** communities with soil physicochemical properties in the five *P. australis* habitats.

To identify potential interactions between bacteria and fungi in the soil of *P. australis*, a co-occurrence network of microbial communities was constructed (Figure 5). Several important topological properties of the microbial community co-occurrence network were listed in Table 3. The network exhibited non-random co-occurrence patterns, as indicated by the R^2^ of the power-law higher than 0.7. Moreover, the values of the average clustering coefficient (ACC) and average path length (APL) were much higher than those of random network, indicating that the network has ‘small world’ properties. In the soil microbial co-occurrence network of *P. australis,* bacteria and fungi mainly showed positive synergistic interactions, as indicated by the 95.17% positive edges (Supplementary Table S2). In terms of the co-occurrence network composition, 86.6% of the nodes were affiliated with Proteobacteria (46.4%), followed by Chloroflexi (14.4%), Bacteroidetes (9.5%), Gemmatimonadtes (8.2%), and Acidobacteria (8.1%) (Figure 5B). Correspondingly, the distribution of links in the co-occurrence network conformed to the distribution of nodes. Bacteria-involved links accounted for 97.3%, whereas fungi–fungi links only accounted for 0.5%. Among these links, those derived from Proteobacteria and Chloroflexi with other phyla were predominant, accounting for 69.3 and 27.1%, respectively (Figure 5B).

**Table 3:** Topological properties of the co-occurrence network of microbial community.

	Network indices	Microbial network
Empirical network	Nodes	745
	Edges	1800
	*R*^2^ of power-law	0.954
	ACC	0.352
	APL	8.16
	Diameter	29
	Modularity	0.745
	Density	0.006
	Positive edges(%)	95.17
	Negative edges(%)	4.83
Random network	APLr	4.36 ± 0.02
	ACCr	0.01 ± 0.00

ACC, Average Clustering Coefficient; APL, Average Path Length; Subscript r, property of the Erdos–Renyi random network. A total of 1,000 random runs were performed to obtain the standard deviations of the APLr and ACCr.

The topological properties of individual nodes were determined based on *Zi-Pi* analysis (Figure 5C). According to the within-module connectivity (*Zi*) and among-module connectivity (*Pi*) of individual nodes, nodes were divided into four classifications: peripherals (*Zi* < 2.5, *Pi* < 0.62), connectors (*Zi* < 2.5*, Pi* > 0.62), module hubs (*Zi* > 2.5, *Pi* < 0.62), and network hubs (*Zi* > 2.5, *Pi* > 0.62) [Olesen et al., 2007]. Among these four classifications, connectors, module hubs, and network hubs were identified as generalists, and were also considered to be keystone taxa that maintain the balance of co-occurrence networks (Montoya et al., 2006). In the present study, only 2.3% of the nodes were affiliated with generalists in the co-occurrence network. Among these 24 generalists, 23 nodes were affiliated with bacteria, and 1 node was affiliated with fungi. It is worth noting that most bacterial keystones were rare taxa, whereas the only fungal keystone was abundant rare taxa (Supplementary Table S3).

### Relationships Among Microbial Community Composition, Function, Co-occurrence Network, and Soil Physicochemical Properties

**Figure 6:**
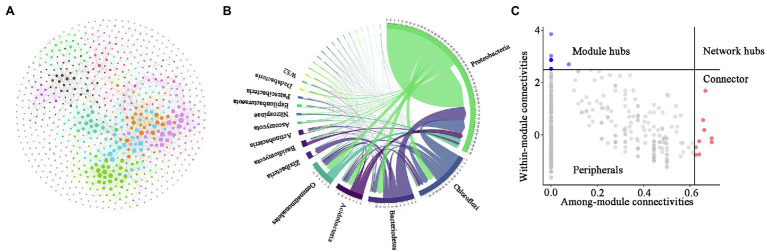
Characteristics of the microbial community co-occurrence network. Network analysis reveals the relationships among fungal operational taxonomic units (OTUs) and bacterial OTUs **(A)**. CIRCOS plot showing the distribution of connections **(B)**. *Zi-Pi* plot revealing the keystone OTU of thee microbial co-occurrence network **(C)**. Each connection represents a strong correlation (*r* > 0.8, *p* < 0.05) between the connected OTUs. The colour of each node shows the module that the node belongs to. The size of nodes is proportional to their connections.

**Figure 7:**
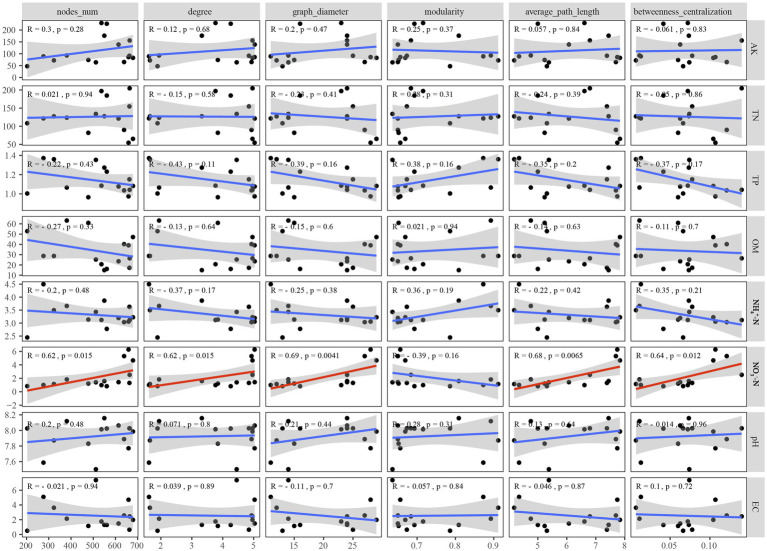
Correlations between soil physicochemical properties and network level topological characteristics.

To better understand the causes of changes in microbial communities, we conducted a Procrustes analysis of the relationships between the microbial community composition and soil physicochemical properties (Figure 6). The results revealed that bacterial community composition was significantly correlated with soil characteristics (*M*^2^ = 0.731, *p* < 0.05), whereas the fungal community composition was not significantly affected by soil characteristics (*M*^2^ = 0.893, *p >* 0.05). The measured soil physicochemical properties explained 23.14% of the variation in the bacterial community and 19.70% of the variation in the fungal community, as indicated by CCA (Figure 6). Among these physicochemical properties, AK, OM, and ${\mathrm{NH}}_{4}^{+}-N$ played important roles in affecting the bacterial community composition, while ${\mathrm{NO}}_{3}^{-}-N$ and OM played vital roles in shaping the fungal community. Pearson correlation analysis showed that the Shannon indices of bacterial and fungal communities were significantly negatively correlated with ${\mathrm{NH}}_{4}^{+}-N$ and ${\mathrm{NO}}_{3}^{-}-N$ , respectively (*p* < 0.05; Supplementary Figure S3). However, analysis of microbial community function and soil physicochemical properties revealed that bacterial (*M*^2^ = 0.623, *p* < 0.05) and fungal (*M*^2^ = 0.665, *p* < 0.05) community functions were significantly associated with soil physicochemical properties. Moreover, among all soil properties detected, ${\mathrm{NO}}_{3}^{-}-N$ was the only soil physicochemical property that significantly affected the topological properties of the co-occurrence network of the microbial community, but the network modularity was not affected (Figure 7).

## Discussion

### Responses of Bacterial and Fungal Community Compositions to *Phragmites australis* Habitats

Generally, microbial community composition in the Yellow River Delta is affected by changes in soil properties and vegetation characteristics caused by human disturbance and habitat heterogeneity (Bickel and Or, 2020; Chi et al., 2021; Wu et al., 2021). Surprisingly, neither bacterial nor fungal ɑ-diversity, such as the Shannon and Chao1 indices, were affected by the habitats with different soil properties (Tables 1 and 2). Similarly, other research conducted in the Yellow River Delta showed the bacterial chao1 index had no significant difference between the supratidal and intertidal habitats of the *Suaeda salsa* community (Liu et al., 2020). In the present study, although soil TN and different forms of N ( ${\mathrm{NO}}_{3}^{-}-N$ and ${\mathrm{NH}}_{4}^{+}-N$ ) significantly related to the diversity of bacteria and fungi, differences in these nutrients between habitats did not cause significant differences in microbial diversity (Supplementary Figure S3). In terms of the composition of microbial communities, our results showed that bacterial communities were significantly affected by different *P. australis* habitats, whereas fungal communities were not (Figures 2C,D). This result can be explained by the relationships between soil physicochemical properties and microbial communities, as indicated by Procrustes analysis (Figures 5A,B), that is, soil physicochemical properties drove variation in bacterial communities. Liu et al. (2020) highlighted that compared with bacterial diversity, bacterial community composition was more sensitive and responds more quickly to a variety of soil physicochemical properties. Another study evaluating global surface soil microbial communities also supported our conclusion that soil physicochemical properties had a greater impact on bacteria than fungi, as geographic distance played an important role in fungal community construction (Bahram et al., 2018). Furthermore, Sweeney et al. (2021) revealed that plant phylogenetic relatedness and plant traits were important factors that determine rhizosphere fungal community composition. The number of samples in this study may also be a factor contributing to the fungal community composition being unaffected by *P. australis* habitat. Compared to the low intra-group variation in bacterial communities, the greater intra-group variation in fungal communities may result in fungal communities that were unaffected by *P. australis* habitat. Therefore, consideration should be given in subsequent studies to increasing the number of samples in order to reduce the potential limitations posed by the small number of samples. Combined with the above results, we suggested that plant types in a community were more likely to cause differences in the soil fungal community than soil physicochemical properties.

In terms of microbial community composition, the most dominant bacterial phylum was Proteobacteria, which has also been reported in previous studies conducted in the Yellow River Delta (Gao et al., 2021; Wu et al., 2021). Various substrate utilisation capabilities, energy acquisition methods, strong environmental adaptability, and rapid growth have been considered to help Proteobacteria become the predominant bacterial phylum in the Yellow River Delta (Bernhard et al., 2005; Mukhopadhya et al., 2012). Moreover, other dominant bacterial phyla have also been reported to play key roles in elemental cycling and energy flow, such as Acidobacteria and Bacteroidetes, which participate in the nitrogen cycle; Acidobacteria and Chloroflexi are the dominant decomposers of OM (Eichorst et al., 2018; Fang et al., 2018; Zhai et al., 2018). The composition of the fungal community was simpler than that of the complex bacterial community. Owing to the strong environmental adaptability and reproductive ability of Ascomycota, its relative abundance was an absolute advantage in each sample, which has also been reported in a variety of habitats, such as pioneer forests and tropical oligotrophic peatlands (Zhong et al., 2018; Morrison et al., 2021).

At the family level, the bacterial community was mainly composed of Methlophagaceae, Desulfobulbaceae, Flavobacteriaceae, Nitrincolaceae, and Marinobacteraceae, which were widely distributed in many coastal wetland soils (Zhang et al., 2012; Risgaard-Petersen et al., 2015; Chen et al., 2020; Yang et al., 2021). Desufobulbaceae, regarded as ‘cable bacteria’, was involved in the formation of a centimetre-deep suboxic zone in marine sediment through their role in the electrical couple of sulfide oxidation and oxygen reduction (Risgaard-Petersen et al., 2015). Methylotroph Methlophagaceae, an obligate that uses C1-compounds contaning methanol and methylamine produced by methane monooxygenase bacteria, had a relatively high relative abundance in all samples, indicating the adaptation of the bacteria to the unique environment of the coastal wetland (Neufeld et al., 2008; Moussard et al., 2009). Moreover, the high content of ${\mathrm{NO}}_{3}^{-}-N$ in S1 enabled Nitrincolaceae with strong heterotrophic nitrate reduction to ammonium ability to be recruited in large amounts, which was beneficial to the growth of plants (Yang et al., 2021). Thus, the significant differences among functional bacteria caused by different habitats significantly affected bacterial community function (Supplementary Figure S2; Figure 4). As the amount of data in the fungal classification database is much less than that of bacteria, multiple fungi with high relative abundances have not been effectively classified at the family level, such as unclassified_kingdom_Fungi, unclassified _class_Sordariomycetes, and unclassified_phylum_Ascomycota (Figure 3D). Among fungal families with good classification information, salt tolerance, and adaptation to anaerobic environments, fungal phyla Pleosporaceae, Aspergillaceae, and Cladosporiaceae were found to have high relative abundances in all habitats, being widely distributed despite the different degrees of saline-alkali soil in the Yellow River Delta (Yang and Sun, 2020). Moreover, Glomeraceae, which is involved in mycorrhizal formation, was largely enriched in S1, S2, and S4, but was rarely detected in other habitats, indicating the existence of mycorrhiza in the root system of *P. australis*; however, it was affected by different habitats (Ban et al., 2017).

### Soil Microbial Functions in Different Habitats

As ecosystem decomposers, bacteria and fungi directly participate in biogeochemical cycles and energy fluxes, and changes in their compositions and functions directly and indirectly affect ecosystem function (Treseder and Lennon, 2015; Laforest-Lapointe et al., 2017; Wagg et al., 2019). The results of PERMANOVA and Procrustes analysis revealed that bacterial community function was affected by habitat (*p* < 0.05) and soil physicochemical factors (*M*^2^ = 0.623, *p* < 0.05) (Figure 4A). Soil physicochemical properties can significantly affect the function of the bacterial community by altering the composition of the bacterial community or influencing the activity of bacteria, such as the nitrogen fixation capacity of Trichodesmium spp., which was affected by the content of nitrogen (Mulholland and Capone, 2000). Among the Kyoto Encyclopedia of Genes and Genomes (KEGG) metabolic pathway groups at level 1, metabolism was the most predominant process, especially carbohydrate metabolism, amino acid metabolism, cofactor, and vitamin metabolism, which were the top three in total relative abundance, indicating high C and N turnover rates in coastal wetlands, which was also reported by Zhang et al. (2016) and Liu et al. (2020). Carbohydrate metabolism, involving the degradation of hemicellulose and cellulose, can release various inorganic substrates and readily available substances to promote microbial and plant growth (López-González et al., 2015). It is not surprising that the prevalence of amino acid metabolism was due to the developed root system containing rhizomes of *P. australis,* which can release root exudates containing amino acids into the soil (Coops and Velde, 1995; Weidenhamer et al., 2013). Microbial metabolism of amino acids not only enhances their growth and development but also promotes plant growth by releasing inorganic nitrogen. Moreover, vitamins can be involved in carboxylation reactions, fatty acid metabolism, and amino acid metabolism as enzyme cofactors, and their metabolism can maintain metabolic homeostasis (Petri et al., 2019). Overall, the high relative abundance of metabolic pathways suggested that bacteria play a key role in the elemental cycle and biotransformation of OM.

The FUNGuild prediction results showed that the soil fungal community function of *P. australis* was not significantly different among the five habitats but was significantly affected by soil physicochemical properties. Considering that the effect of habitat on microbial community function is mainly through biotic (e.g., plant community) and abiotic factors (e.g., environmental factors), we speculate that the effect of the *P. australis* community on fungal community function was stronger than the effect of the environment on fungal community function (Waters et al., 2017). Saprophytic fungi are the main degradators of plant and animal residues, and their high relative abundance in the soil of *P. australis* habitats indocated that fungi play key roles in the degradation of OM and elemental cycling. As another relatively abundant fungal trophic mode, arbuscular mycorrhizal fungi (AMF) can form symbionts with roots to promote nutrient absorption and stress tolerance of plants (Berruti et al., 2016; Mathur et al., 2019). However, AMF were enriched only in S1, S2, and S4, indicating that, compared with 80% common infection, the proportion of *P. australis* roots and AMF forming symbiosis was still relatively low (Figure 4D). Moreover, a variety of plant and animal pathogens were classified in all habitats with high relative abundance, indicating that wetland habitats of *P. australis* were repositories of fungal pathogens (Chen et al., 2020). Similarly, the large intra-group variation caused by the small number of samples could be a potential reason for the lack of significant effects of *P. australis* habitat on fungal community function. In general, FUNGuild is a functional classification system based on existing studies and is limited by the amount of information in the fungal comparison database; as such, a more realistic understanding of fungal community function requires further exploration.

### Topological Features of the Co-occurrence Network and Their Controlling Factors

Soil microorganisms do not exist as individual species but form complex ecological networks through positive, negative, and neutral interactions, including but not limited to competition, commensalism, and mutualism (Faust et al., 2015; Graham et al., 2016). Menezes et al. (2017) highlighted the importance of a comprehensive understanding of the interactions between fungi and bacteria to improve soil ecosystem services. In the present study, we constructed intra- and inter-kingdom co-occurrence networks based on bacterial and fungal OTUs. The results showed that intra-kingdom interactions (bacteria, bacteria, fungi, and fungi) form more easily than inter-kingdom (bacteria–fungi) interactions, indicating that microorganisms were more susceptible to the influence of intra-kingdom microorganisms (Duan et al., 2021). Moreover, considering that the positive correlations between microbes were determined by their positive ecological interactions, such as commensalism and mutualism, it is not surprising that most interactions were positive because the degradation of OM requires the cooperation of various microorganisms, especially recalcitrant OM containing cellulose, hemicellulose, and lignin (Wang et al., 2016). Recently, microbial co-occurrence networks that were more inclined toward intra-kingdom symbiosis have been reported in a variety of habitats, such as the rhizosphere and mountainous areas (Duan et al., 2021; Qian et al., 2021).

A keystone microbial taxa is a highly connected taxonomic group in the co-occurrence network that significantly influence microbial community structure and function irrespective of their abundance across time and space (Banerjee et al., 2018). According to the *Zi-Pi* classification principle of keystone taxa and the identification principle of abundant/rare OTUs, we found far more bacteria than fungi in the identified keystone taxa, indicating that bacteria play a more critical role in maintaining the structural stability of the co-occurrence network (Zhu et al., 2021a). Furthermore, most of these bacterial keystone taxa were rare species, whereas the only fungal keystone identified was an abundant species. Indeed, a growing number of studies have highlighted the key role that rare species play in maintaining microbial community structure (Power et al., 1996; Xu et al., 2021; Zhu et al., 2021b). Generally, rare taxa of bacteria are highly active, as indicated by higher 16S rRNA/rRNA genes, and can increase rapidly under changing environments (Campbell et al., 2011). In addition, the lack of sensitivity of rare species to abiotic changes caused by the fact that these rare species are neither opportunistic nor take advantage of the reduction of dominant species may be the reason why rare species become the majority of keystones (Kurm et al., 2019). Notably, most rare bacterial species and abundant fungal species as keystone taxa that played important roles in maintaining the stability of intra-kingdom microbial co-occurrence networks have also been reported in cadmium-contaminated soil, but whether or not this phenomenon was random requires further study (Xu et al., 2021).

An increasing number of studies have focused on the relationships among soil physicochemical properties, ecological functions, and topological properties of microbial co-occurrence networks. Ma et al. (2016) revealed the importance of geographic distance, climatic factors, and soil physicochemical properties in network topological features. Zhang et al. (2018) emphasised that soil physicochemical properties, especially pH, available nitrogen, and available Mg, were significantly related to the topological properties of the microbial network. Moreover, the multifunctionality of the soil ecosystem was positively correlated with the network complexity index of the number of edges and nodes (Qiu et al., 2021). In this study, Spearman’s correlation analysis was conducted to explore the impact of soil physicochemical properties on the key topological properties of the network. Among the detected soil physicochemical properties, ${\mathrm{NO}}_{3}^{-}-N$ was the only one that could affect the topological properties of the microbial network (Figure 7). This was consistent with the high amino acid metabolism capacity of soil bacterial communities that dominate the co-occurrence network in different *P. australis* habitats (Figure 4C). Dissimilatory nitrate reduction to ammonium bacteria, such as Nitrincolaceae, was widely distributed in wetlands and can effectively transform the existing form of nitrogen, and thus affects the interactions between microorganisms (Zhao et al., 2020). Furthermore, as the only network topological feature unaffected by soil physicochemical properties, modularity may be more susceptible to the influence of phylogenetic relatedness, as indicated by the classification information of microorganisms with the same modularity being more similar (Hu et al., 2017).

## Conclusion

Our study showed that different *P. australis* habitats have no significant effect on the richness and diversity of soil bacterial and fungal communities. In terms of microbial community composition, the fungal community was more stable than the bacterial community, as indicated by the bacterial community being significantly affected by habitat, in contrast with the fungal community. Similarly, the predicted function of bacterial community was to respond to the variation in habitats, whereas that of the fungal community was not. Additionally, combined with the result that both predicted functions of bacterial and fungal communities were significantly associated with soil physicochemical properties. The dominant predicted function of soil bacterial and fungal communities was to participate in the degradation of OM (e.g., carbohydrate metabolism, amino acid metabolism, and saprotrophic function). Network analysis revealed that microorganisms mainly participated in the formation of complex interaction networks through positive interactions. In terms of maintaining network structure, most rare species were identified as keystone taxa; the ${\mathrm{NO}}_{3}^{-}-N$ content was an essential factor significantly related to the topological properties of the microbial network. Together, we provided reliable basic ecological knowledge for further study on microbial composition and function of wetland soil.

## Data Availability Statement

The datasets presented in this study can be found in online repositories. The names of the repository/repositories and accession number(s) can be found at: https://www.ncbi.nlm.nih.gov/, PRJNA807493 and PRJNA807797.

## Author Contributions

PZ: conceptualization, writing—original draft, and data curation. SY: data curation and formal analysis. YW: data curation. YR: data curation. XY: writing—review and editing. LW: funding acquisition and methodology. WG: funding acquisition, project administration, and writing—review and editing. All authors contributed to the article and approved the submitted version.

## Funding

This work was financially supported by the Nation Natural Science Foundation of China [31970347], Key Technology Research and Development Program of Shandong [2021CXGC010803].

## Conflict of Interest

The authors declare that the research was conducted in the absence of any commercial or financial relationships that could be construed as a potential conflict of interest.

## Publisher’s Note

All claims expressed in this article are solely those of the authors and do not necessarily represent those of their affiliated organizations, or those of the publisher, the editors and the reviewers. Any product that may be evaluated in this article, or claim that may be made by its manufacturer, is not guaranteed or endorsed by the publisher.

## Footnotes

^1^ www.i-sanger.com
