# Supplementary material for: Shifts in Soil Microbial Community Composition, Function, and Co-occurrence Network of Phragmites australis in the Yellow River Delta
Source: Front Microbiol. 2022 Jul 19;13:858125. doi: 10.3389/fmicb.2022.858125 (PMC9344067; doi:10.3389/fmicb.2022.858125)
Supplement: Supplementary file 1 [file Data_Sheet_1.docx]

**Shifts in soil microbial community composition, function and co-occurrence network in different** ***Phragmites australis communities* of Yellow river Delta**

Pengcheng Zhu^1^, Shuren Yang^1,2^, Yuxin Wu^1^, Yuning Ru^1^, Yihang Hou^1^, Xiaona Yu^1^, Lushan Wang^3,*^, Weihua Guo^1,*^.

1. Institution of Ecology and Biodiversity, School of Life Science, Shandong University, Qingdao 266237, PR China
2. State Key Laboratory of Microbial Technology, Shandong University, Qingdao 266237, PR China


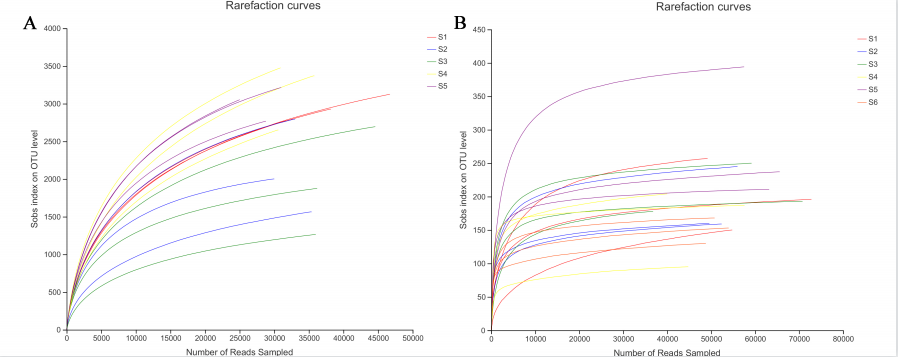


Fig.S1 Rare curves of bacterial (A) and fungal (B) OTUs.


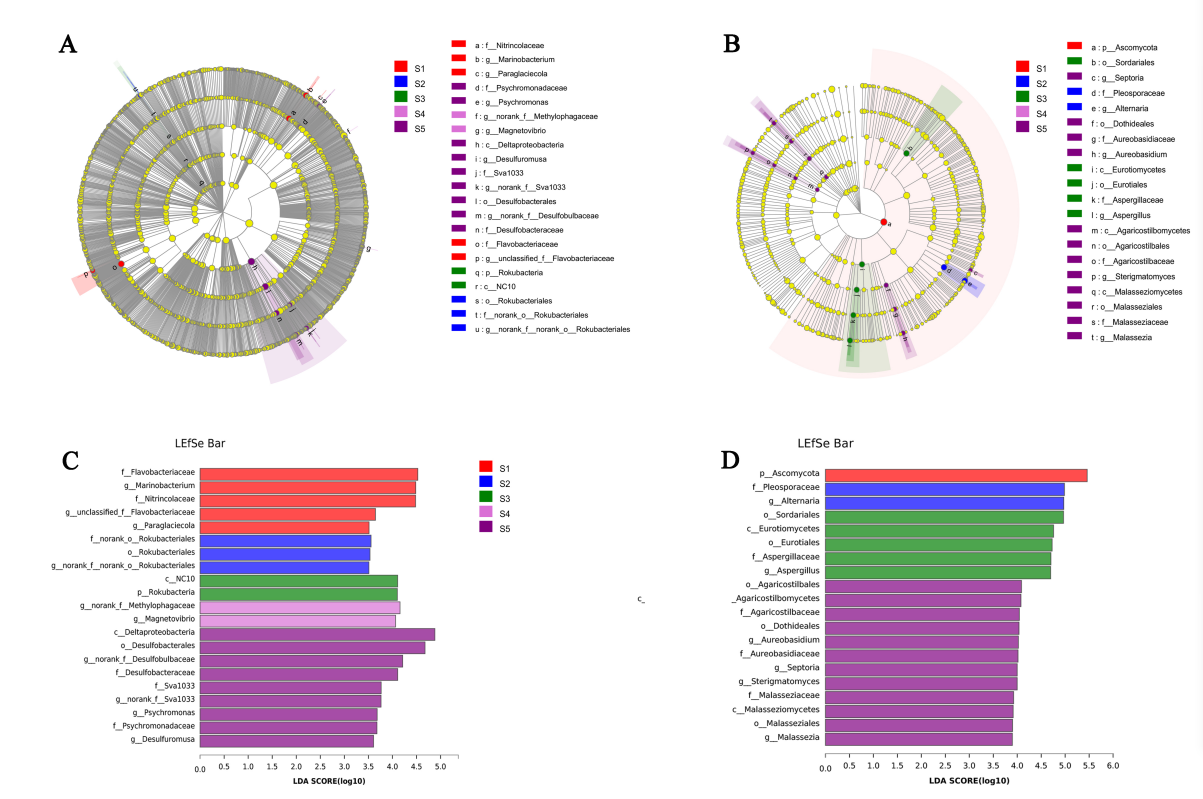


Fig. S2 The differential phylogenetic distribution of bacteria (A) and fungi (B) in different habitats. Biomarker bacteria (C) and fungi (D) with liner discriminant analysis score > 3.5 in different habitats. Nodes of different colors represent microorganisms that play an important role in the group represented by the color, and yellow represents unimportant microorganisms.


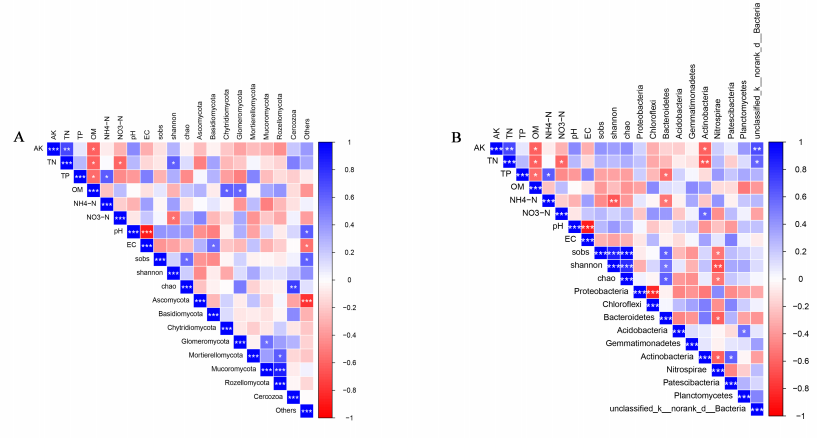


Fig.S3 The relationship between bacterial (A) and fungal (B) phyla and soil physicochemical property.

Table S1 The information about different ***P. australis*** habitats.

| Sampling point | Longitude | Latitude | Description |
| --- | --- | --- | --- |
| S1 | 118°58′49″E | 37°43′48″N | Located near saltwater; community contains Suaeda salsa |
| S2 | 119°9′49″E | 37°45′31″N | Community contains Tamarix chinensis |
| S3 | 118°59′22″E | 37°43′38″N | Close to the tidal barrier; plants were short |
| S4 | 119°5′18″E | 37°45′38″N | Close to the fresh water; plants were tall |
| S4 | 119°2′20″E | 37°49′59″N | Intertidal zone, community contains Spartina alterniflora |

Table S2 The composition of links in soil microbial co-occurrence network.

| Type | total | positive | negative |
| --- | --- | --- | --- |
| Bacteria-Bacteria | 1751 | 1593 | 69 |
| Bacteria-Fungi | 40 | 32 | 8 |
| Fungi-Fungi | 9 | 9 | 0 |

Table S3 The category information of keystone

| TYPE | Kingdom | Phylum | Class | Order | Family | Genus | taxa |
| --- | --- | --- | --- | --- | --- | --- | --- |
| Module hubs | Bacteria | Acidobacteria | Subgroup_6 |  |  |  | rare |
| Module hubs | Bacteria | Acidobacteria | Thermoanaerobaculia | Thermoanaerobaculales | Thermoanaerobaculaceae | Subgroup_10 | rare |
| Module hubs | Bacteria | Bacteroidetes | Bacteroidia | Cytophagales | Cyclobacteriaceae | Marinoscillum | rare |
| Module hubs | Bacteria | Bacteroidetes | Bacteroidia | Cytophagales | Cyclobacteriaceae |  | rare |
| Module hubs | Bacteria | Bacteroidetes | Bacteroidia | Chitinophagales | Saprospiraceae |  | rare |
| Module hubs | Bacteria | Bacteroidetes | Bacteroidia | Cytophagales | Cyclobacteriaceae | Catalinimonas | rare |
| Module hubs | Bacteria | Chloroflexi | Anaerolineae | SBR1031 | A4b |  | rare |
| Module hubs | Bacteria | Chloroflexi | Anaerolineae | SBR1031 | A4b |  | rare |
| Module hubs | Bacteria | Chloroflexi | Chloroflexia | Thermomicrobiales | AKYG1722 |  | rare |
| Module hubs | Bacteria | Chloroflexi | Anaerolineae | SBR1031 | norank_SBR1031 |  | rare |
| Module hubs | Bacteria | Dadabacteria | Dadabacteriia | Dadabacteriales | norank_Dadabacteriales |  | abundance |
| Module hubs | Bacteria | Gemmatimonadetes | Gemmatimonadetes |  |  |  | abundance |
| Module hubs | Bacteria | Gemmatimonadetes | Gemmatimonadetes |  |  |  | rare |
| Module hubs | Bacteria | Proteobacteria | Gammaproteobacteria | EPR3968-O8a-Bc78 |  |  | rare |
| Module hubs | Bacteria | Proteobacteria | Deltaproteobacteria | Desulfuromonadales | Sva1033 |  | rare |
| Module hubs | Bacteria | Proteobacteria | Alphaproteobacteria | Rhizobiales | Xanthobacteraceae |  | rare |
| Module hubs | Bacteria | Proteobacteria | Gammaproteobacteria | Betaproteobacteriales | Rhodocyclaceae | Azoarcus | abundance |
| Module hubs | Bacteria | Proteobacteria | Alphaproteobacteria | Rhodospirillales | Magnetospiraceae | Magnetospira | rare |
| Module hubs | Bacteria | Proteobacteria | Alphaproteobacteria | Sphingomonadales | Sphingomonadaceae | Sphingorhabdus | rare |
| Module hubs | Bacteria | Proteobacteria | Deltaproteobacteria | RCP2-54 |  |  | rare |
| Connectors | Bacteria | Proteobacteria | Gammaproteobacteria |  |  |  | rare |
| Connectors | Bacteria | Proteobacteria | Alphaproteobacteria | Rhodobacterales | Rhodobacteraceae |  | rare |
| Connectors | Bacteria | Proteobacteria | Alphaproteobacteria |  |  |  | rare |
| Module hubs | Eukaryota | Ascomycota | Sordariomycetes |  |  |  | abundance |
